# Incidence of Pressure Ulcers in Adults Hospitalized in Intensive Care Units in Colombia

**DOI:** 10.17533/udea.iee.v43n2e12

**Published:** 2025-07-25

**Authors:** Olga L. Cortés, Juan C. Villar, Yudy A. Rojas, Skarlet M. Vásquez, Jessica Ruiz-Sandoval, Claudia P. Becerra, Víctor Herrera, Researchers from PENFUP-2 Colombia Participating Hospitals

**Affiliations:** 1 Nurse, PhD. Associate Researcher. Email: ocortes@cardioinfantil.org. Corresponding author. https://orcid.org/0000-0002-9023-7479 Fundación Cardioinfantil Colombia ocortes@cardioinfantil.org; 2 MD. MSc. PhD. Research and Professor. E-mail: jvillar@unab.edu.co, jvillarc@lacardio.org. https://orcid.org/0000-0002-7047-7299 Fundación Cardioinfantil Colombia jvillar@unab.edu.co; 3 Nurse, Specialist. E-mail: jrojas@cardioinfantil.org. https://orcid.org/0000-0002-5939-3457 Fundación Cardioinfantil Colombia jrojas@cardioinfantil.org; 4 RN, MSc. Professor. E-mail: svasquez196@unab.edu.co. https://orcid.org/0000-0003-2552-9819 Universidad Autónoma de Bucaramanga Colombia svasquez196@unab.edu.co; 5 RN MSc. Researcher. E-mail: jruizs@lacardio.org. https://orcid.org/0000-0001-5458-8346 Fundación Cardioinfantil Colombia jruizs@lacardio.org; 6 RN, MSc. Researcher. E-mail: cbecerra@lacardio.org. https://orcid.org/0000-0003-4778-1150 Fundación Cardioinfantil Colombia cbecerra@lacardio.org; 7 MD, PhD. Professor. E-mail: vicmaher@uis.edu.co. https://orcid.org/0000-0002-6295-1640 Universidad Autónoma de Bucaramanga Colombia vicmaher@uis.edu.co; 8 Fundación Cardioinfantil - Instituto de Cardiología, La Cardio. Bogotá DC, Colombia. Fundación Cardioinfantil Colombia; 9 Universidad Autónoma de Bucaramanga, Colombia Universidad Autónoma de Bucaramanga Universidad Autónoma de Bucaramanga Colombia

**Keywords:** incidence, pressure ulcer, nursing, critical care, incidencia, úlcera por presión, enfermería, cuidados críticos., incidência, úlcera por pressão, enfermagem, cuidado intensivo.

## Abstract

**Objective.:**

To estimate the incidence of pressure ulcers (PU) in hospitalized adults and its relationship with prevention practices in adult intensive care units (ICU) in Colombian hospitals.

**Methods.:**

This was a multicenter prospective cohort study in 31 non-COVID-19 ICUs from 11 hospitals in Colombia, including 1543 patients without ability to move, but with healthy skin, admitted consecutively upon admission to ICU. The primary outcome was the incidence of PU per 1,000 days of hospital stay.

**Results.:**

The participants were mostly men (57.5%), with mean age of 59±18 years and body mass index of 25.5±4.6 kg/m^2^. The study observed 120 PU in 17 063 days of hospital stay, the majority were in the sacral region (60.0%) and heels (10.8%). Overall incidence was of 7.03 (95%CI 5.9-8.41) by 1000 days-patient. The PU incidence rate was double in ICUs of public hospitals than in private hospitals [Incidence Rate Ratio (IRR) = 2.00; 95%CI: 1.30 to 3.01]. The risk of pressure ulcers was lower in hospitals that had skin-care group (IRR = 0.38; 95%CI: 0.25 to 0.58), used dressings [IRR = 0.66; 95%CI: 0.45 to 0.95] and support surfaces [IRR = 0.37; 95%CI: 0.24 to 0.59] in their preventive care practices.

**Conclusion.:**

Much variability was noted in the PU incidence among the hospitals observed. However, Grade I and II ulcers and located in the sacral region continue having the highest incidence, according with global reference data. Hospital preventive care patterns reported a series of interventions administered in ICU that can be related with the risk of PU.

## Resumen

**Objetivo.:**

Estimar la incidencia de úlceras por presión (UPP) en adultos hospitalizados y su relación con las prácticas preventivas en unidades de cuidados intensivos (UCI) de adultos de hospitales colombianos.

**Métodos.:**

Estudio multicéntrico de cohorte prospectiva en 31 UCIs no Covid-19, de 11 hospitales de Colombia. Se incluyeron 1543 pacientes sin capacidad para movilizarse, pero con piel sana, admitidos consecutivamente al momento de la admisión en UCI. El desenlace primario fue la incidencia de UPP por mil días-estancia hospitalaria.

**Resultados.:**

Los participantes eran en su mayoría hombres (57.5 %), con media de edad 59±18 años y un índice de masa corporal de 25.5±4.6 kg/m^2^. Se observaron 120 UPP en 17 063 días estancia hospitalaria, la mayoría fueron en región sacra (60.0 %) y en talones (10.8 %). La incidencia general fue de 7.03 (IC95% 5.9-8.41) por 1000 días-paciente. La tasa de incidencia de UPPs fue el doble en las UCIs de hospitales públicos que en los privados [Razón de Tasas de Incidencia (RTI) = 2.00; IC95%: 1.30 a 3.01]. El riesgo de UPP fue menor en hospitales que tenían grupo de cuidado de la piel (RTI = 0,38; IC95%: 0,25 a 0,58), usaban apósitos [RTI = 0.66; IC95%: 0.45 a 0.95] y superficies de apoyo [RTI = 0,37; IC95%: 0,24 a 0,59] en sus prácticas de cuidado preventivo.

**Conclusión.:**

Se observó una gran variabilidad en la incidencia de UPP entre los hospitales observados. Sin embargo, las úlceras Grado I y II y ubicadas en la región sacra continúan siendo las de mayor incidencia, acorde con datos referentes a nivel mundial. Los patrones de cuidado preventivo hospitalario reportaron una serie de intervenciones administradas en UCI que pueden relacionarse con el riesgo de UPP.

## Resumo

**Objetivo.:**

Estimar a incidência de úlceras de pressão (UPP) em adultos hospitalizados e sua relação com práticas preventivas em unidades de terapia intensiva (UTI) adultas em hospitais colombianos.

**Métodos.:**

Um estudo de coorte prospectivo multicêntrico foi conduzido em 31 UTIs não Covid-19 de 11 hospitais na Colômbia. Um total de 1.543 pacientes com mobilidade prejudicada, mas pele saudável, admitidos consecutivamente no momento da admissão na UTI, foram incluídos. O desfecho primário foi a incidência de UPPs por 1.000 dias de internação hospitalar.

**Resultados.:**

Os participantes eram em sua maioria homens (57.5%), com idade média de 59 ± 18 anos e índice de massa corporal de 25,5 ± 4.6 kg/m ^2^ . 120 UPPs foram observadas em 17.063 dias de internação hospitalar, a maioria das quais na região sacra (60.0%) e calcanhares (10.8%). A incidência geral foi de 7.03 (IC 95% 5.9-8.41) em 17.063 pacientes-dia. A taxa de incidência de úlceras de pressão foi duas vezes maior em UTIs de hospitais públicos do que em hospitais privados [Razão da Taxa de Incidência (IRR) = 2.00; IC 95%: 1.30-3.01]. O risco de úlceras de pressão foi menor em hospitais que tinham um grupo de cuidados com a pele (IRR = 0.38; IC 95%: 0.25-0.58), usavam curativos [IRR = 0.66; IC 95%: 0.45-0.95] e usavam superfícies de suporte [IRR = 0.37; IC 95%: 0.24-0.59] em suas práticas de cuidados preventivos.

**Conclusão.:**

Houve uma grande variabilidade na incidência de úlceras de pressão entre os hospitais observados. No entanto, úlceras de grau I e II localizadas na região sacra continuam apresentando a maior incidência, de acordo com dados globais. Os padrões de cuidados preventivos hospitalares relataram uma série de intervenções administradas na UTI que podem estar relacionadas ao risco de úlceras de pressão.

## Introduction

Pressure ulcers (PU), also known as recumbent ulcers or pressure injuries represent one of the adverse events of greater impact on the quality of life of hospitalized patients, on their relatives, and on costs for health systems.^1^ These lesions are located in the skin and underlying tissue, and are produced as consequence of increased external pressure on some bony prominence, and constitute a complication during hospitalization in high-risk patients, especially those bedridden.^1^^,^^2^ The complications associated with these lesions can produce increased morbidity and mortality during the hospital stay.^2^ According to the Agency for Healthcare Research and Quality (AHRQ), the number of individuals affected by PU exceeds 2.5-million yearly.^3^^,^^8^ A systematic literature review of the global disease burden 1990-2019^9^^,^^10^ reported a slight trend in the decrease of the incidence and of the global prevalence [reduction of 10.6%, 95%CI 8.7% to 12.3% in incidence and of 10.2% in prevalence, 95%CI 8.2% to 11.9%), respectively.^9^^,^^10^ Likewise, reports indicate by 2019 a standardized prevalence by age of 11.3 (95%CI 10.2 to 12.5), incidence of 41.8 (95%CI 37.8 to 46.2) and 1.7 [95%CI 1.2 to 2.2] years of life with a disability (YLD) for every 100-thousand inhabitants.

Critically ill patients hospitalized in ICU constitute one of the groups most-vulnerable to developing these lesions, which are associated with states of greater fragility related with their state of health, which worsens with immobility and prolonged periods in bed.^11^^,^^12^ The global incidence of PU in ICUs varies between 3.3% and 53.4%,^10^ prevalence between 13.1 % and 45.5%,^10^ and hospital mortality associated with complications due to PU between 23% and 27%.^12^^-^^15^ In Colombia (2019) the incidence rate in ICUs has been estimated between 7.0% and 26.9%.^14^ The determining factors in adult ICU include variables, like age [ ≥70 years OR: 2.14 95%CI 1.27-3,6; presence of comorbidities, such as type-II diabetes mellitus [OR 5.58 95%CI 1.83-18.7], admission to ICU due to trauma and diagnosis of sepsis, respectively [OR: 15.9 95%CI 3.7-68; OR: 2.89 95%CI 1.16-7.22], and use of vasopressor medications [vasopressin OR: 4.81 95%CI 1.66-13.92; noradrenalin OR: 3.68 95%CI 1.12-12.16], and ventilator therapy >7 hours [OR 23.0 95%CI 6.42-86.0].^12^^,^^14^

At hospital level, a series of preventive measures have been implemented within the framework of the organization of policies and PU prevention programs in intensive care in hospitals throughout the world. Pressure ulcer reduction has been reported with the implementation of some protective measures, like postural changes/repositioning [OR: 0.45 95%CI 0.21-0.97], use of dynamic surfaces [OR: 0.87 95%CI 0.81-0.94], use of dressings on sacral region [absolute reduction of PU of 9.2%, 95%CI: 2.3% to 16%], and implementation of group care measures or packages [0.37 95%CI 0.24 -0.57].^12^ In this sense, it is required for hospitals and clinics to guide their care plans towards improving safety and quality to reduce the onset of these events in patients at risk. Existence of a prevention policy that includes using clinical practice guides (CPG) with effective evidence-based interventions, structuring of a skin-care group that guides preventive care, assessment of indicators, and the provision of human and material resources are determining strategies in preventing PU in ICU and other hospital services.^12^^,^^13^^,^^16^

Although in Colombia prior studies have reported PU prevalence and incidence during hospitalization, such have not characterized the incidence of these events in critical care, where these rates are possibly higher.^17^ Moreover, these studies lack the description of the characteristics of the practice environments and institutional prevention policies related with human, physical, and organizational resources, and of the conventional practices used to prevent these lesions in ICU^18^^-^^21^, which are key to understand the tendency of this problem and the planning of nursing interventions in hospital prevention. Consequently, this study’s overall objective was to determine the incidence of PU in adults hospitalized in ICUs of hospital in Colombia. It also sought to describe standardized preventive care practices and their relationship with the PU incidence in these units.

## Methods

Design. A secondary analysis was performed of the cohort of the control group of the cluster randomized clinical trial called “Evaluation of two levels of repositioning frequency in reduction of pressure ulcers PENFUP-2” (Registry clinialtrials.gov NCT04604665)^22^ whose objective was to evaluate the effectiveness of the high frequency of repositioning in reducing PUs in 22 hospitals-ICUs with respect to the frequency of postural change according to conventional care in the incidence of PU. The study was approved by the ethics committees of the 11 hospitals. The patients from the control group were observed during their conventional care; they were recruited in 31 ICUs (9 medical, 9 mixed, 6 surgical, 4 neurological, 3 coronary) in 11 high-complexity hospitals located in Bogotá (*n* = 5), Bucaramanga (*n* = 2), Medellín (*n* = 2), Barranquilla *(n* = 1), and Manizales *(n* = 1) between 1^st^ April 2021 and 30^th^ March 2023. The hospitals included had to have adult ICU service (≥18 years), offer specialized care to critically ill patients with any type of care emphasis, and have a minimum of 10 beds. The study included intermediate care units, burnt patient care units, and units to manage patients with COVID-19 or mixed including patients with COVID-19. With respect to patients, these were eligible if upon admission they had intact skin, *i.e.* without PU (no degree of ulcer), as well as complete inability to mobilize and requirement for hospitalization ≥24 hours. Patients with device- or adhesive-associated ulcers (MARSI) or incontinence dermatitis were not included. PENFUP-2 was approved by the ethics committees of all the participating hospitals, likewise, all the patients signed an informed consent accepting voluntary participation.

Procedures. The study’s lead researcher in each hospital evaluated the eligibility of patients that consecutively were hospitalized in the ICUs during the observation period. The patients were included with informed consent previously signed by their relatives. Monitoring since their recruitment was daily until the primary outcome occurred, that is, development of the first pressure ulcer (PU) or mobility began autonomously, or discharge from the la ICU or death. The PUs were classified as Grade I, II, III, or IV according to that established by the National Pressure Ulcer Advisory Panel (NPUAP)^23^ and the National Group for the Study and Advice on Pressure Ulcers and Chronic Wounds - Spain (GNEAUP, for the term in Spanish),^24^ and in keeping with the definition by the Pressure Ulcer Prevention Guide in Colombia.^13^ The events were validated by the quality staff in each hospital. Furthermore, for each patient, sociodemographic and clinical information on admission (comorbidities) was obtained; the risk of PU was determined using the Braden scale, as well as the occurrence of complications during hospitalization, including those associated with repositioning, time of hospital stay, and reason for discharge from the ICU. With regard to the standardized prevention strategies, each hospital's typology was determined (public-university or private-university), as well as the existence of a skin-care group, and specifically for ICUs, the ratio of the number of nurses per patient, the number of nurses specialized in intensive care, and implementation of the following preventive care practices: standardized use of special pressure management surfaces (SPMS), moisturizing cream, preventive dressings, fatty acids, and repositioning frequency. The nursing professional leader of each ICU and the study collaborators received training to verify the patients’ eligibility criteria, as well as to collect admission and follow up data, which were entered into electronic formats using the REDCap program^25^ and subject to permanent audit by the study’s central committee. 

Statistical analysis. The sample size estimated for the control group in the PENFUP-2 study *(n* =1,650) permitted reaching precision between ±1.0% and ±1.5% for PU incidences between 5.0% and 10.0%, respectively. The sample was described by estimating means (standard deviations [SD]) and medians (interquartile range [IQR]) in the case of continuous variables with and without normal distribution, respectively, according to the Kolmogorov-Smirnov test; while discrete variables were summarized through absolute and relative frequencies (%). The hypotheses of differences among the groups of patients who did or did not develop PU regarding the distribution of the continuous variables were evaluated using Student’s t test (Wilcoxon for those without normal distribution) and for the discrete variables through the Chi-squared test or, alternatively, Fisher’s exact test. Finally, PU incidence rates were estimated (per 1,000 patients-day) and their respective confidence intervals of 95% (95%CI), as well as the incidence rate ratios (IRR) and 95%CI among the levels of the independent variables at individual level (patients) and conglomerate level (hospitals). A two-tailed statistical significance level of 5% was defined. All the analyses were performed with the Stata version 12 statistical program.

## Results

The work evaluated 1543 patients, mostly of male sex (57.5%), with mean age of 59.0 years (SD = 18.0 years) and body mass index (BMI) of 25.7 kg/m^2^ (SD = 4.6 kg/m^2^). The most prevalent comorbidities on admission to the ICUs were cardiovascular *(n* = 867; 56.2%), respiratory (*n* = 361; 23.4%), metabolic, such as type-II diabetes mellitus (n = 298; 19.3%), and neurological (*n* = 229; 14.8%). Regarding risk of PU, 47.6% and 45.2% of the patients were classified as high and very high risk, respectively, according with the Braden scale ^28^**(**Table 1).

**Table 1:** Characteristics, condition, and repositioning frequency of patients in ICU

Characteristics	Total *N = 1543*	With PU *n = 120*	Without PU *n = 1423*	***p-*value**
Age (years), mean [SD]	59.3 (19.1)	59.0 (18.8)	59.3 (19.1)	0.825
Men, *n* (%)	887 (57.5)	72 (60.0)	815 (57.3)	0.562
Body mass index (kg/m^2^), mean [SD]	25.7 (4.6)	25.5 (4.6)	25.7 (4.6)	0.439
Comorbidities*, *n* (%)				
Cardiovascular	867 (56.2)	63 (52.5)	804 (56.5)	0.392
Respiratory	361 (23.4)	33 (27.5)	328 (23.1)	0.271
Type-II Diabetes	298 (19.3)	31 (25.8)	267 (18.8)	0.060
Neurological	229 (14.8)	12 (10.0)	217 (15.3)	0.120
Renal	211 (13.7)	22 (18.3)	189 (13.3)	0.123
Cancer	148 (9.6)	10 (8.3)	138 (9.7)	0.624
Peripheral vascular disease	58 (3.8)	6 (5.0)	52 (3.7)	0.458
Malnutrition	43 (2.8)	6 (5.0)	37 (2.6)	0.125
**Braden scale, *n* (%)**				
Low	31 (2.2)	1 (0.8)	30 (2.4)	0.228
Medium	69 (4.9)	2 (1.7)	67 (5.2)	
High	665 (47.6)	61 (50.8)	604 (47.3)	
Very high	631 (45.2)	56 (46.7)	575 (45.1)	
Hospitalization ICU				
Duration of stay in ICU/days, mean [SD]	12.6 (13.8)	20.5 (18.3)	11.9 (13.1)	<0.001
**Complications during hospitalization in ICU, *n* (%)**				
Sepsis	254 (16.5)	41 (34.2)	213 (14.9)	<0.001
Cardiovascular	244 (15.8)	28 (23.3)	216 (15.2)	0.019
Respiratory	215 (13.9)	32 (26.7)	183 (12.9)	<0.001
Kidney failure	167 (10.8)	26 (21.7)	141 (9.9)	<0.001
Bleeding	163 (10.6)	21 (17.5)	142 (9.9)	0.010
Neurological	85 (5.5)	9 (7.5)	76 (5.3)	0.319
Delirium	107 (6.9)	15 (12.5)	92 (6.5)	0.012
Death	330 (21.4)	33 (27.5)	297 (20.9)	0.089
Repositioning frequency, mean (SD)	76.0 (85.2)	75.0(107.7)	76.1(83.0)	0.475
**Repositioning frequency, *n* (%)**				
Every 2 hours	943 (61.1)	37 (30.8)	906 (63.7)	<0.001
Every ≥3 hours	600 (38.9)	83 (69.2)	517 (36.3)

*Patients may have more than one comorbidity. SD = Standard deviation

Of all the patients, 120 (7.8%) developed at least one PU during follow up for an incidence rate of 7.0 (95%CI: 5.9 - 8.4) per 1,000 days-patient (range between participating hospitals 0.0 - 41.1, Table 2). No differences were observed in age, sex, BMI, comorbidities, or risk of PU among patients who developed or not PU; nevertheless, incident cases had longer times of hospital stay: 20.5 versus 11.8 days. In addition, it was noted that the group of patients who developed PU had a higher frequency of complications, including cardiovascular (23.3% versus 15.2%), respiratory (26.7% versus 12.9%), renal (21.7% versus 9.9%), bleeding (17.5% versus 9.9%), delirium (12.5% versus 6.5%), and sepsis (34.2% versus 14.9%), but not mortality, during the stay in ICU compared with the group of patients who did not develop PU. Regarding preventive repositioning frequency throughout the stay in ICU no statistically significant difference was observed between the groups that did or did not develop PU (76.0 versus 75.0, respectively); however, the incident group was characterized with a high periodicity of low-frequency position changes every 4, 6, and 8 hours (69.2%) compared with the group that did not have events, which presented a high frequency of mobility every 2 h (63.7%).

**Table 2:** Incidence of pressure ulcers per hospital

Hospital	*n*	PU	Days-patient	Incidence rate* (95%CI)
1	150	0	1250	0.0(0.0-0.0)
2	86	1	1305	0.8(0.1 to 5.4)
3	150	2	1004	2.0(0.2 to 8.0)
4	150	11	3562	3.1(1.7 to 5.6)
5	107	4	1048	3.8(1.4 to 10.2)
6	150	12	2268	5.3(3.0 to 9.3)
7	150	7	1162	6.0(2.9 to 12.6)
8	150	13	1766	7.4(4.3 to 12.7)
9	150	21	1511	13.9(9.1 to 21.3)
10	150	27	1652	16.3(11.3 to 23.8)
11	150	22	535	41.1(27.1 to 62.5)
Total	1543	120	17 063	7.0(5.9 to 8.4)

* Per 1,000 patients-days.

According with their location, PUs were observed more frequently in the sacral region (4.22 per 1,000 patient-days; 95%CI: 3.3 - 5.3), followed by the heels (0.76 per 1,000 patient-days; 95%CI: 0.4 - 1.3) and the face (0.52 per 1,000 patient-days; 95%CI: 0.2 - 1.0 (Table 3**)**. Regarding the severity of the lesions, such was distributed, thus: 33.3%, 62.5%, 2.5%, and 1.7% as grade I, II, III, and IV, respectively.

**Table 3:** Incidence of 120 pressure ulcers according to area of location

Body area	Events (*n* = 120)	Incidence rate*	(95%CI)
Sacrum	72	4.22	3.30 to 5.31
Heel	13	0.76	0.40 to 1.30
Face	9	0.52	0.24 to 1.00
Elbow	6	0.35	0.13 to 0.76
Occipital	5	0.32	0.09 to 0.68
Auricle	5	0.32	0.09 to 0.68
Scapula	3	0.17	0.03 to 0.51
Shoulder	3	0.17	0.03 to 0.51
Chest	2	0.11	0.01 to 0.42
Iliac Crest	1	0.05	0.00 to 0.32
Ankle	1	0.05	0.00 to 0.32

All the hospitals included were tier III level of complexity: 9 private-university and 2 public-university. With respect to preventive care practices, all the hospitals had an institutional policy to prevent PUs and standardized the use of moisturizing cream. Additionally, of the 11 hospitals, 9 had a skin group, 9 implemented the use of support surfaces, 6 used anti-bedsore mattresses, 6 used preventive skin dressings, 4 used fatty acids, and 7 had a periodicity standard of repositioning every two hours. Regarding the distribution of professional nurses per patient in ICU, in two (18.2%) hospitals the ratio ranged between 1:1 and 1:2, in three (27.3%) it was 1:3, and in six (54.5%) it was higher than 1:3 (Table 4).

**Table 4:** Description of the standardized prevention strategies in hospitals and incidence of pressure ulcers

Standardized interventions	Events	Days-patient	Incidence rate (95%CI) ^*^	Ratio of incidence rate (95%CI)
Type of hospital				
Private-University	86	14,249	6.0 (4.9 to 7.5)	1.00
Public-University	34	2,814	12.1 (8.6 to 16.9)	2.00 (1.30 to 3.01)
Institutional skin care monitor group				
Yes	85	14,762	5.8 (4.7 to 7.1)	0.38 (0.25 to 0.58)
No	35	2,301	15.2 (10.9 to 21.2)	1.00
Nurse-patient ratio				
1:1-1:2	7	2,412	2.9 (1.4 to 6.1)	1.00
1:3-1:4	28	8,183	3.4 (2.4 to 4.9)	1.18 (0.50 to 3.19)
≥1:5	85	6,468	13.1 (10.6 to 16.3)	4.52 (2.10 to 11.60)
Anti-bedsore mattresses				
Yes	97	10,576	9.2 (7.5 to 11.2)	2.59 (1.63 to 4.27)
No	23	6,487	3.5 (2.4 to 5.3)	1.00
Mobility protocol/24 h				
Every 1-2 hours	37	11,599	3.2 (2.3 to 4.4)	1.00
≥3 hours	83	5,464	15.2 (12.2 to 18.8)	4.76 (3.19 to 7.22)
Use of preventive dressings				
Yes	61	10,440	5.8 (4.5 to 7.5)	0.66 (0.45 to 0.95)
No	59	6,623	8.9 (6.9 to 11.5)	1.00
Protection with fatty acids				
Yes	55	5,575	9.9 (7.6 to 12.8)	1.74 (1.19 to 2.54)
No	65	11,488	5.7 (4.4 to 7.2)	1.00
Use of support surfaces				
Yes	94	15,480	6.1 (4.9 to 7.4)	0.37 (0.24 to 0.59)
No	26	1,583	16.4 (11.2 to 24.1)	1.00

* Per 1000 patient-days.

The incidence rate of PUs was double in ICUs of public-university hospitals than in private-university hospitals (IRR=2.00; 95%CI: 1.30 - 3.01) but 62% lower in those that had a group to monitor skin care (IRR=0.38; 95%CI: 0.25 - 0.58) (Table 3). The use of preventive dressings [IRR= 0.66; 95%CI: 0.45 to 0.95] and support surfaces [IRR=0.37; 95%CI: 0.24 to 0.59] were associated to lower PU incidence (34% and 63% less risk, respectively), use of anti-bedsore mattresses, protection with fatty acids, and repositioning periodicity ≥3 hours were associated with a greater incidence of ulcers. Furthermore, a dose-response relationship was observed in terms of the nurse-patient ratio and the incidence of PU: a greater number of patients per nurse was associated with a greater incidence of lesions (Table 4).

## Discussion

This study report, to our knowledge, the first prospectively planned estimate of the incidence of pressure ulcers in the intensive care units of a diverse group of high-complexity hospitals in Colombia. In addition, it explores its relationship with the institutional preventive care practices therein. The reported incidence of 7.03% [95%CI 5.8%-8.41%] of the first ulcer acquired during the stay in the participating ICUs of the hospitals included in the study provides a reference for hospital administrators and caregivers in said care scenario, represents the lower limit of the data previously reported in Colombia (2019) (7.0% and 26.9%).^14^ The location of greater frequency in the sacral region is quite similar to that of most studies of this type and does not indicate that probably preventive care must be aimed at caring for this body area. The study described that private-university hospitals and implementation of preventive measures in ICU, such as having a continuous skin care monitoring group at institutional level, use of dressings, and use of support surfaces showed lower incidence of PU. Likewise, it is indicated that the high number of patients per caregiver, application of low frequencies of repositioning, and use of anti-bedsore mattresses and fatty acids increased the onset of PU.

Our estimate gains additional value due to the difficulty of comparing with other studies, given that these report the accumulated rate and not the incidence rate. The incidence rate (IR) includes as denominator the time of stay of each individual in ICU during a given time and accumulated rate (AR) bears in mind only the number of individuals exposed, assuming that patients have the same hospital stay and, thus, the same risk. A systematic literature review of hospitals in Brazil included nine studies, which reported IR with great variation (5.6% to 65.3%).^26^ Something similar happened in our study, which found hospitals with rates from 0.0% or 14%, reaching a maximum of 41%. This variability by hospitals, in the case of Brazil, according to the authors could be related with their being or not in a developed part of the country. In our study, the variability could be because different blocks of preventive care are used in one or another hospital, some with better follow-up standards in private hospitals, which - in turn - are university teaching centers, have the best resources, and have the best quality of care.^27^

The onset of PU in critically ill patients continues being a problem of great impact that affects specially patients in critical state throughout the world. The variability in the incidence of PU depends to a large extent on multiple factors, not only demographic like old age but those related with the patient’s state of health and frailty and dependence levels that lead to developing greater complications that prolong the hospital stay.^28^ Although in our study only patients with history of type-II diabetes had greater incidence of PU, anticipated identification of other comorbidities that can compromise the circulatory system and alter cardiac output and, thus, tissue perfusion is a determining factor in risk assessment upon admission to these critical care services and in planning individualized preventive care.^29^

Patients who developed cardiorespiratory complications in the ICU, as well as sepsis, renal failure, delirium, and bleeding, had a higher proportion of PU. Low cardiac output and low oxygenation lead to tissue hypoperfusion, which requires management with vasoconstrictor medications and ventilator use, care measures that further increase the risk of tissue hypoperfusion injury.^30^ Moreover, these types of complications lead to longer hospital stay, and - consequently - to longer periods of bed rest, immobility and, thus, the appearance of PU.^30^ In this study, patients with PU had longer times of hospital stay compared with those without PU [20.5 days vs 11.8], (mean difference 8.6 days [95%CI 6.12-11.21]). Critically ill patients with prolonged hospital stay require aid for their repositioning in bed due to their functional deconditioning. Also, immobility in bed or the administration of low repositioning frequency is a determining factor of PU in these patients in ICU.^34^ Our study shows similar results as those reported elsewhere, evidencing that hospitals that implemented a high repositioning frequency of every 2 h had lower incidence of PU.^30^^,^^31^

Our study reported a higher frequency of Grade II (62.5%) and Grade I (33%) ulcers. Overall, studies reporting incidence and prevalence do not report Grade I lesions, likely due to the difficulty in being properly identified, which may lead to classification bias. Training of the staff involved in ICU on differentiating the types of lesion and existence of a skin care group and institutional quality allowed for the control of potential bias associated with lesion classification.^32^ This training was conducted according to indications from the health system in our country^13^ and by following international recommendations for better accuracy of the types of ulcers in our study. As in other studies, inclusion of Grade I injuries, although not of mandatory report to the Social Protection System, must be reported early to stop their progression to more advanced stages. In addition, identification of a high number of Grade II ulcers indicates that Grade I lesions were not identified on time and, consequently, these progressed to this stage, as may have occurred in our study, given the high incidence of Grade II ulcers.^33^

The site of greatest occurrence of ulcers was the sacral region, a result similar to most studies conducted on ICU patients and patients hospitalized in other hospital services.^31^ These lesions are related to prolonged permanence of patients at risk in supine-semi-recumbent position (30-45° inclination angle) and low repositioning frequency that apparently reduces ventilator-associated pneumonia.^34^ Likewise, sacral lesions are much more prevalent because their onset is associated with the proximity of the perianal area, its humidity, and organic fluids.^30^

Standardized preventive care patterns on the appearance of PU were explored in our study. All the hospitals in the study reported having a preventive care policy and - additionally - used moisturizing cream for skin care, general practices whose effectiveness is still unknown. A lower incidence of ulcers was identified in private-university hospitals, those with a hospital skin care monitoring group, with a repositioning standard every 2 h, with a nurse-patient ratio from 1:1 to 1:2, those that used preventive dressings, and used support surfaces. These care patterns have been standardized following some of the International Clinical Practice Guidelines;^23^^,^^24^ but the adjustment of the recommendations is mostly based on weak positive evidence or there is not yet sufficiently robust evidence (based on clinical trials). Furthermore, although we relate the use of certain care patterns that appear in the CPG, the interventions are not applied generally to all patients in ICU, which indicates that further and better quality research is still warranted on the prescription of certain preventive care. Exposure assessment, as done in this study, exploring skin care strategies recorded at the institutional level, but with the outcome (PU) recorded at the individual level may carry a margin of error.^35^ Thus, it is fundamental to assess the interventions conducted in each patient in each center to establish estimates closer to reality.^35^

In conclusion, the PU incidence estimated in the 11 hospital ICUs participating in Colombia was 7.0 (5.9 to 8.4). A large variability in reported rates was observed among hospitals, from having no PU to reporting high rates, such as 16.3 (11.3 - 23.8) and 41.1 (27.1 - 62.5). The most-frequent location of this lesion was the sacral region (4.22, 95%CI 3.30-5.31), followed by the heels (0.76, 95%CI 0.40-1.30) and face (0.52, 95%CI 0.24-1.00), and a high frequency of injury severity between I and II (95.8%). By relating the incidence rate with the care patterns reported by each hospital, it was identified that private hospitals had a lower incidence of PU compared with public hospitals.

Among the hospital care patterns, applied to ICU patients that reported low rates of PU, included having a skin care monitoring group compared to hospitals that do not have it; having a mobilization or repositioning plan every 2 h; maintaining a nurse-patient ratio between 1:1 and 1:2 and between 1:3 and 1:4, compared with hospitals that have a ratio ≥ 1:5. Low PU rates were also observed in hospitals that have implemented the use of support surfaces and preventive dressings in their care plan. Among the hospital care patterns that reported high rates of PU, there is the use of anti-bedsore mattresses, along with use of repositioning or position changes protocols ≥ every 3 h and use of fatty acids.

Political and clinical implications. The findings herein are crucial given that they are strongly related with structuring of nursing preventive care plans reflected in the patient’s risk and safety outcomes. The findings permit identifying the occurrence of PU in individuals with intact skin and the PU incidence report allows our greater understanding of the tendency of this situation in hospital health while indicating the guidelines that must be improved in health and the interventions that must be implemented for better prevention of these events. Although our study observed several strategies in the diverse hospitals about prevention related with nursing care and a specialized support group to implement these strategies in all the hospitals included, support of preventive practices should be extended to all the hospitals caring for population at risk. Similarly, it is key to advance in research on the effectiveness of preventive interventions to produce quality scientific evidence. Updating and improving the National Clinical Practice Guide related to preventing these events must be considered by the Ministry of Social Protection, which oversees hospital safety.

Strengths and limitations of the study. The principal strength of this study is that it was based on a multicenter prospective cohort design of several hospitals in Colombia, including critically ill patients with high risk of developing PU, but with intact skin. Also, this study evaluated the incidence rate according to each patient’s hospital stay. Finally, this study registered the preventive strategies implemented in the hospitals evaluated and explored their association with the incidence rate, nevertheless, studies, such as clinical trials, are required to confirm the effectiveness of the interventions described in our study. Although the study explored the possibility of including hospitals from remote zones of the country, it is likely that in said hospitals skin care is less structured and the incidence higher than that reported in our study. The incidence rate of pressure ulcers is an indicator that reflects the quality of care in hospitals and healthcare centers. Variability in implementing preventive care may be affected by administrative factors. Thus, the results presented herein do not reflect the incidence of all the hospitals in Colombia, which may be underestimated. The results indicate that a greater application of preventive measures is required in our hospitals. The results obtained may indicate that these preventive measures may possibly not apply to all the patients, and further evidence of the effectiveness and benefit of these interventions is needed to justify their use.

Researchers from PENFUP-2 Colombia Participating Hospitals. Yohana Milena Garzón; Mabel Rocío Parra Santos ; Luisa Fernanda Patarroyo Albarracín; Ana Ruby Vásquez Bejarano; Ángela Viviana Aguirre Salazar; Carolina Bedoya Vásquez; Daniela Correa García; Andrea Rosas Santana; María Isabel Pulgar; Harvein Herrera Paredes; Silvia Elena Cuervo Dávila; Erika Carolina Cruz Suarez; Paula Andrea Montoya Pérez; Oswaldo Juan Arroyave Cardona; Juan Camilo Gómez Ortiz ; Maribel Esparza; Cristina Useche; Janeth Moreno; Vilma Ángel Cruz; Hernán Herrera; Julián Andrés Caballero Bernal; Sandra Patricia Pulido Barragán; Carlos Harvey Jaimes Pabón; Ana María Henao Bedoya; Manuela Londoño Céspedes; Ana María Gallego Marín; Sandra Gutiérrez Olivares; Paola Murcia González; Margarita María Pérez Pérez; Tatiana Isabel Valbuena Mancipe; Jenny Yasmin Salas Pérez; Rocío del Pilar Cabezas Daca; Alejandro Ortiz; Hna. Claudina Pineda Bustos; Diana Carolina Silva Henao; Gina Marcela Ibañez Sanguino; Karen Cecilia Vergara Castellón; Nelson Enrique Zapata, Diana Arango; Gladys Castro Pinto; Lina Rocío Duarte Parra; Leidy Estefanía Cabarcas Silva; Liliana Mora; Luisa Cristancho; Lyda Zoraya Rojas Sánchez; Johanna Astrid Navarro Peña; Diana Díaz Mass; Claudia Vásquez Soto; Kelly Fernández Ahumada.
